# An Electrochemical, Low-Frequency Seismic Micro-Sensor Based on MEMS with a Force-Balanced Feedback System

**DOI:** 10.3390/s17092103

**Published:** 2017-09-13

**Authors:** Guanglei Li, Junbo Wang, Deyong Chen, Jian Chen, Lianhong Chen, Chao Xu

**Affiliations:** Institute of Electronics, Chinese Academy of Sciences, Beijing 100190, China; liguanglei13@mails.ucas.edu.cn (G.L.); dychen@mail.ie.ac.cn (D.C.); chenjian@mail.ie.ac.cn (J.C.); chenlianhong15@mails.ucas.edu.cn (L.C.); xuchao16@mails.ucas.edu.cn (C.X.)

**Keywords:** electrochemical seismic sensor, microfabrication, negative feedback, repeatability

## Abstract

Electrochemical seismic sensors are key components in monitoring ground vibration, which are featured with high performances in the low-frequency domain. However, conventional electrochemical seismic sensors suffer from low repeatability due to limitations in fabrication and limited bandwidth. This paper presents a micro-fabricated electrochemical seismic sensor with a force-balanced negative feedback system, mainly composed of a sensing unit including porous sensing micro electrodes immersed in an electrolyte solution and a feedback unit including a feedback circuit and a feedback magnet. In this study, devices were designed, fabricated, and characterized, producing comparable performances among individual devices. In addition, bandwidths and total harmonic distortions of the proposed devices with and without a negative feedback system were quantified and compared as 0.005–20 (feedback) Hz vs. 0.3–7 Hz (without feedback), 4.34 ± 0.38% (without feedback) vs. 1.81 ± 0.31% (feedback)@1 Hz@1 mm/s and 3.21 ± 0.25% (without feedback) vs. 1.13 ± 0.19% (feedback)@5 Hz@1 mm/s (n_device_ = 6, n represents the number of the tested devices), respectively. In addition, the performances of the proposed MEMS electrochemical seismometers with feedback were compared to a commercial electrochemical seismic sensor (CME 6011), producing higher bandwidth (0.005–20 Hz vs. 0.016–30 Hz) and lower self-noise levels (−165.1 ± 6.1 dB vs. −137.7 dB at 0.1 Hz, −151.9 ± 7.5 dB vs. −117.8 dB at 0.02 Hz (n_device_ = 6)) in the low-frequency domain. Thus, the proposed device may function as an enabling electrochemical seismometer in the fields requesting seismic monitoring at the ultra-low frequency domain.

## 1. Introduction

A seismic sensor functions as a velocity sensor or an accelerometer that senses the ground vibration of the earth, which is widely used in the field of earthquake monitoring, resource exploration, and ocean bottom observation [1,2,3]. The studies of long-period surface waves and slow seismic processes require better seismic data in the low-frequency domain [4,5]. Seismic sensors include moving-coil seismic sensors [6,7,8], fiber-optic seismic sensors [9,10], pendulum seismometers [11,12], and micro-electromechanical system (MEMS) accelerometers [13,14,15]. Conventionally, seismologists have detected low-frequency seismic vibrations by increasing the quality of the inertial mass or decreasing the equivalent spring coefficient, leading to the decrease of the natural frequency, which, however, renders the conventional seismic sensors bulky and vulnerable. Compared to other seismometers, the electrochemical seismic sensors, which is only used for the detection of low seismic signals, are featured with high performance in the low-frequency domain, enabling the measurements of long-period surface waves and slow seismic processes [16,17,18].

The first prototype of the electrochemical seismic sensor was based on “solion,” which was initially developed in the 1950s by the US Navy, targeting the detection of low-frequency acoustic waves [19,20,21,22]. Significant improvements of “solion” were realized in Russia where the electrochemical seismic sensors, which were called molecular electronic transducers in the 1990s, were proposed based on four Pt meshes as electrodes insulated by three porous dielectric ceramic spacers [23,24]. However, the conventional mesh weaving approach and ceramic sintering technologies cannot fabricate electrodes with high repeatability leading to issues of low yield and high cost.

To resolve these issues, the electrochemical seismic sensors based on MEMS technologies were introduced by He et al. [25,26,27], which improved the consistency and decreased the cost significantly [27]. However, the proposed electrochemical seismic micro-sensors still suffered from limited working bandwidth; thus, their applications in ocean bottom seismometers were compromised. 

In this paper, a force-balanced negative feedback system was proposed to extend the working bandwidth of the MEMS-based electrochemical seismometers, which, at the same time, retains the characteristics of high repeatability. In addition, the microfabrication of the sensing electrodes was improved to enhance production yield. The structure of this paper is organized as follows. In Section 2, the structure and the working principle of the MEMS electrochemical seismic sensor with a negative feedback system are described. Section 3 introduces the fabrication and assembling of the MEMS electrochemical seismic sensor. Section 4 provides the characterization of the developed devices. Section 5 concludes the paper.

## 2. Structure and Working Principle

**Figure 1:**
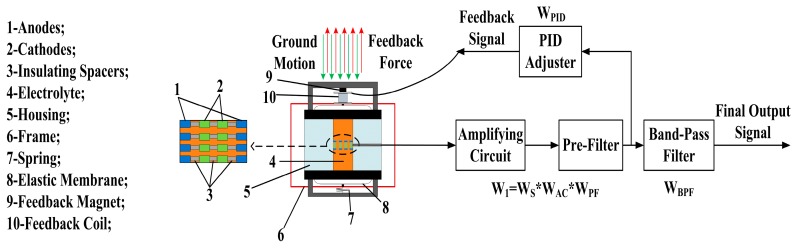
The schematic of the MEMS electrochemical seismic sensor which is composed of a sensing unit including porous sensing electrodes immersed in an electrolyte solution sealed in a plexiglass house by elastic membranes and a feedback unit including a feedback circuit, a feedback magnet, and a feedback coil. In response to external seismic signals, the electrolyte moves opposite to the direction of the external vibration, leading to ion concentration gradients and raw current outputs between the anode–cathode pairs. The raw output signals generated from the sensing unit were processed by the feedback circuit and then applied to the feedback coil, generating feedback forces due to its interactions with the feedback magnet, counterbalancing the movement of the liquid mass.

Figure 1 illustrates the schematic of the MEMS electrochemical seismic sensor with force-balanced negative feedback. The electrochemical seismic sensor consists of a sensing unit and a feedback unit. The sensing unit includes porous sensing electrodes immersed in an electrolyte solution as the liquid mass, which is then sealed by two elastic membranes within a plexiglass house. The porous sensing electrodes fabricated by MEMS technologies are arranged in the anode-cathode-cathode-anode setup to improve the linearity of the output signals. The electrolyte, which is a mixture of iodine (*I*_2_) and potassium iodide (KI), flows through the via-holes in the sensing electrodes. The following reversible electrochemical reactions occur on the surfaces of the anodes and the cathodes, respectively, when a DC bias (0.1–0.3 V) is applied on the electrodes, generating ion concentration gradients between each pair of the anode and the cathode.

(1) $$\mathrm{anode}:\;3I^{-}-2e^{-}\to I_{3}^{-}$$

(2) $$\mathrm{cathode}:\;I_{3}^{-}+2e^{-}\to 3I^{-}$$

where $I_{3}^{-}$ in (1) and (2) is a clathrate combining *I*_2_ with $I^{-}$.

In response to external seismic signals, the electrolyte solution moves opposite to the direction of the external vibration due to the inertial force, which causes the variation of the ion concentration gradients between the anode–cathode pairs. These concentration gradients lead to current outputs, which are proportional to the external seismic signals [25].

The feedback unit composes of a feedback circuit (e.g., a pre-amplification, a pre-filter, and a PID adjuster), a feedback magnet and a feedback coil (see Figure 1). The raw output signals generated from the sensing unit are processed by the feedback circuit and then applied to the feedback coil, positioned within the magnetic field of the feedback magnet. The alternating currents in the feedback coil interact with the magnet field to generate feedback forces, which provides an opposite force to counterbalance the movement of the liquid mass. The transfer function of the closed system is presented in the following form:

(3) $$W=\frac{W_{1}}{1+W_{1}\times W_{PID}}\times W_{BPF}$$

where $W_{1}=W_{s}\times W_{AC}\times W_{PF}$ (*W_S_*, *W_AC_*, and *W_PF_* represent the transfer functions of the sensor, the amplifying circuit, and the pre-filter, respectively), and $W_{PID}$ and $W_{BPF}$ are the transfer functions of the PID adjuster and the band-pass filter, respectively.

In a deep negative feedback ($W_{1}\times W_{PID}\gg 1$) system, Equation (3) can be transformed into the following form:

(4) $$W=\frac{W_{BPF}}{W_{PID}}.$$

As shown in Equation (4), the output signals of the seismic sensor are shaped by $W_{PID}$. The proportional coefficient and the compensation capacitor, two major parameters in the feedback system, can affect the output characterization. 

The pre-filter and the PID adjuster (proportion integration differentiation) in the feedback circuit provides compensations to enhance both the amplitude and the phase margins by adjusting the values of the compensation capacitors. The increases of both amplitude and phase margins enable the system to function in a manner of deep feedback. Additionally, since the feedback force counterbalances the movement of the inertial mass in response to environmental vibrations, the dynamic range of the developed electrochemical seismometer is effectively enlarged.

## 3. Fabrication and Assembling

**Figure 2:**
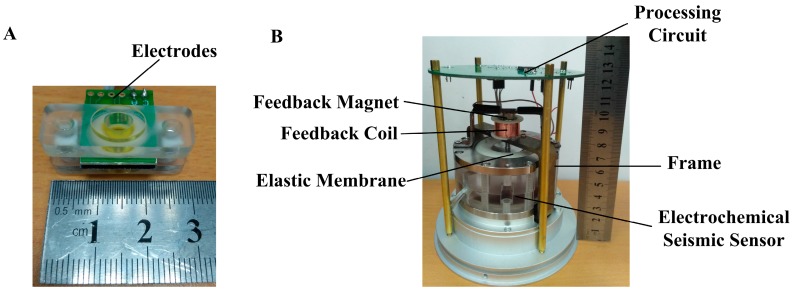
The sensing unit (**A**) and the prototype (**B**) of the MEMS electrochemical seismic sensors with force-balanced negative feedback. The inertial mass composed of the frame, the electrolyte, the membranes, and the feedback coil. The feedback coil and the feedback magnet were not connected together, which may have relative motion between the two components.

The sensing electrodes were fabricated by conventional MEMS technologies including lithography, deep reactive ion etching, thermal oxidation, sputtering, and wire bonding, where detailed processes can be found in previous publications [26]. Then, the integrated electrodes (see Figure 2A) were positioned in a plexiglass tube, which was filled by an electrolyte solution and sealed by two elastic membranes. The molar ratio of *I*_2_ to KI was between 1:50 and 1:100, which ensures that *I*_2_ molecules were fully dissolved in the KI solution in the forms of $I_{3}^{-}$.

Figure 2B introduces the assembled MEMS electrochemical seismic sensor with a feedback system. The feedback coil fabricated with 3D printing was immobilized on the frame, while the feedback magnet was immobilized on the plexiglass housing. Thus, the feedback magnet moves alongside with the seismic sensor, while the feedback coil remains fixed. The feedback circuit including a differential amplification unit, a pre-filter unit, and a PID adjuster was immobilized above the seismic sensors.

## 4. Experimental Characterization

**Figure 3:**
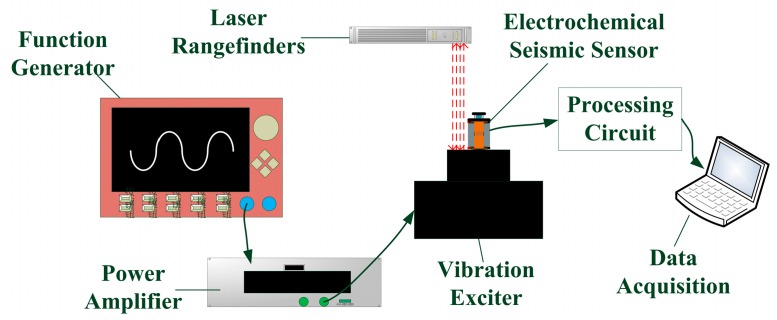
The schematic of the experimental platform where the developed seismic sensor was positioned on a vibration exciter, which is excited electrically (function generation and power amplifier) and detected optically (laser rangefinder).

The characterizations of the MEMS electrochemical seismic sensors were conducted on a modified home-developed platform (see Figure 3), which consists of a function generator, a power amplifier, a laser rangefinder, a vibration exciter, and a data acquisition system. The excited alternative waves were generated from the function generator and amplified by the power amplifier, which were then applied to the vibration exciter to generate vibration signals at a certain frequency. The laser rangefinder was used to measure the velocities of the vibration signals, while the data acquisition system (4472, NI, Austin, TX, USA) was used to measure the output signals.

**Figure 4:**
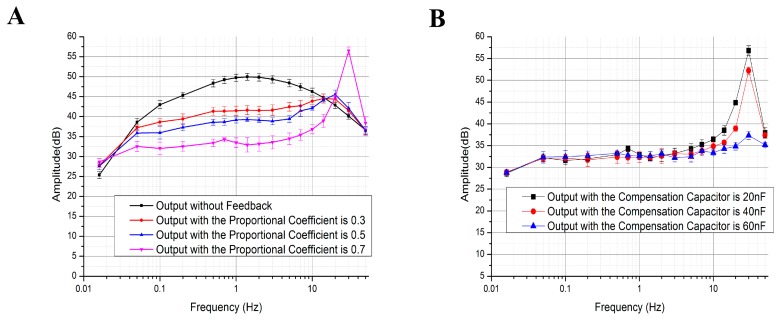
Frequency characterization results of the MEMS electrochemical seismic sensors without feedback, with feedback plus variations in proportional coefficients (**A**) and with feedback plus variations in compensation capacitors (**B**).

Figure 4A shows the frequency characterization results of the MEMS electrochemical seismic sensor without feedback and with feedback plus variations in a proportional coefficient. It was shown that the increase of the proportional coefficient can lead to (1) an increase in the working bandwidth and (2) the possibilities of resonance. Figure 4B shows the frequency characterization results of MEMS electrochemical seismic sensor with different values of compensation capacitors. It was observed that the value of the compensation capacitor had no influence on the output amplitude at the low-frequency domain, which can decrease the possibilities of resonance due to the phase compensation effects of the capacitor. The required working bandwidth can be obtained by adjusting the appropriate values of the proportional coefficient and the compensation capacitor.

**Figure 5:**
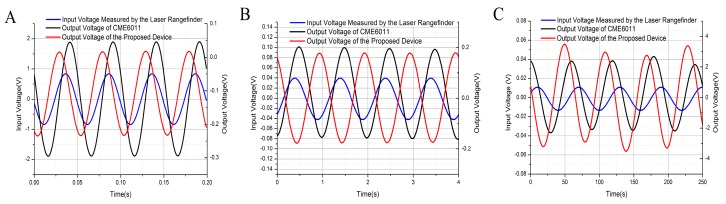
Input voltages measured by the laser rangefinder and the output voltages of CME 6011 and the proposed device @20 Hz (**A**) @0.18 mm/s, @1 Hz@0.17 mm/s (**B**), and @0.016 Hz@3.4 mm/s (**C**).

Figure 5 shows input voltages measured by the laser rangefinder and the output voltages of CME 6011 and the proposed device @20 Hz (A)@0.18 mm/s, @1 Hz@0.17 mm/s (B), and @0.016 Hz@3.4 mm/s (C). It was observed that (1) the output voltages of the proposed device were smaller than that of CME 6011 at 20 Hz due to the working bandwidth limitation of the proposed device, (2) the output voltage of the proposed device was almost the same as that of CME 6011 at 1 Hz, and (3) the output voltage of the proposed device was larger than that of CME 6011 at 0.016 Hz due to the working bandwidth limitation of CME 6011.

**Figure 6:**
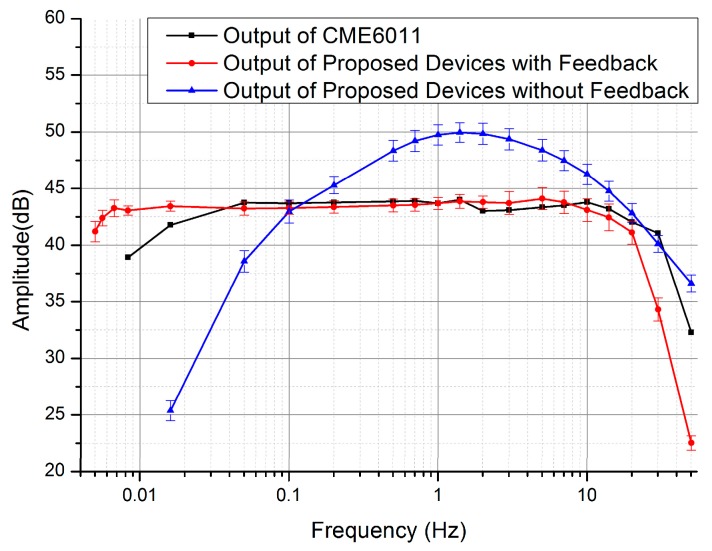
Frequency characterization results of the MEMS electrochemical seismic sensor with and without feedback (n_device_ = 6) in comparison to CME 6011. The amplitude is the output voltage of the electrochemical seismic sensors at the same input velocity and different frequencies.

Figure 6 shows the frequency characteristics of the developed micro seismic sensors with and without the feedback system and CME 6011. More specifically, the electrochemical seismic micro sensors without feedback produced a limited bandwidth at 0.3–7 Hz. By introducing and optimizing the feedback unit, the working bandwidth was extended to 0.005–20 Hz, which was broader than that of CME 6011 with a bandwidth of 0.016–30 Hz.

**Figure 7:**
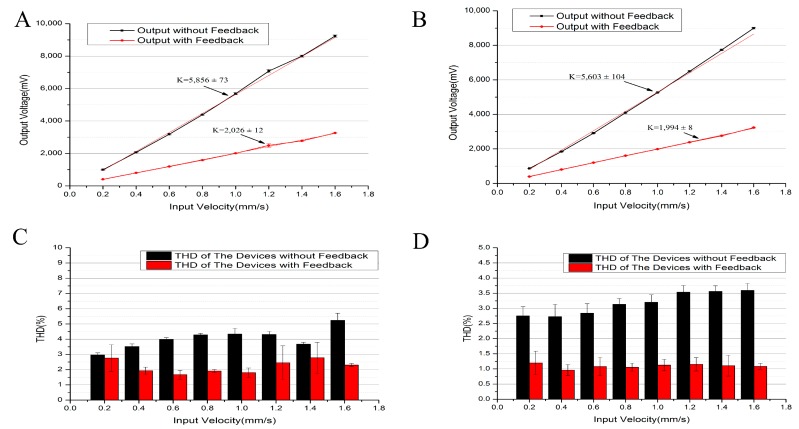
(**A**,**B**) The sensitivity of the MEMS electrochemical seismic sensors with and without feedbacks as a function of input velocities at 1 Hz and 5 Hz, respectively. (**C**,**D**) Total harmonic distortions of the MEMS electrochemical seismic sensors with and without feedbacks as a function of input velocities at 1 Hz and 5 Hz, respectively.

Figure 7 shows the sensitivities of the developed micro seismic sensors with and without the feedbacks, which were quantified as 2026 ± 12 V/m/s vs. 5856 ± 73 V/m/s at 1 Hz and 1994 ± 8 V/m/s vs. 5603 ± 104 V/m/s at 5 Hz (see Figure 7A,B). In comparison to micro seismic sensors without feedback, the micro seismic sensors with feedback produced lower sensitivities due to the feedback forces. However, micro seismic sensors with feedback produced much lower total harmonic distortions compared to the micro sensors without feedbacks (see Figure 7C,D). For instance, the total harmonic distortions (which was calculated by the ratio of the total mean square root of the harmonic wave and the fundamental frequency amplitude.) decreased from 4.34 ± 0.38% (without feedback) to 1.81 ± 0.31% (feedback)@1 Hz@1 mm/s and 3.21 ± 0.25% (without feedback) to 1.13 ± 0.19% (feedback)@5 Hz@1 mm/s. An increase in the amplitude of the output signal lead to an increase in feedback force, which confined the movement of the liquid mass within the linear range, and decreased total harmonic distortions.

**Figure 8:**
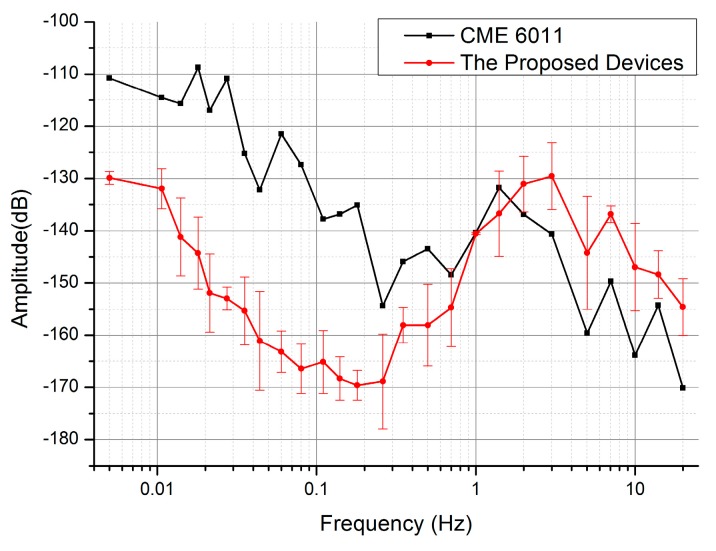
The self-noise power spectrums of the developed MEMS electrochemical seismic sensors with feedback (n_device_ = 6) and the conventional CME 6011 where the developed seismic micro sensors device produced lower self-noise levels than CME 6011 in the frequency domain of 0.01–1 Hz.

The tests of the self-noise levels were also conducted in this platform without inducing vibrations actively. As shown in Figure 8, the self-noise power spectrums of the developed MEMS electrochemical seismic sensors were lower than that of CME 6011 in the frequency domain of less than 1 Hz. More specifically, the self-noise levels were characterized as −165.1 ± 6.1 dB (micro seismic sensors) vs. −137.7 dB (CME 6011) at 0.1 Hz, −151.9 ± 7.5 dB (micro seismic sensors) vs. −117.8 dB (CME 6011) at 0.02 Hz (n_device_ = 6). It was speculated that at a frequency higher than 1 Hz, the noise of the seismic sensor mainly comes from mechanical and environmental noises due to packaging. Thus, the increases in the self-noise levels of the proposed devices may result from imperfect packaging technologies in comparison to commercial seismic sensors.

## 5. Conclusions

A MEMS-based electrochemical seismic sensor with ultra-broad frequency working bandwidth was demonstrated in this paper. The working bandwidth and the self-noise level of the developed device were characterized with feedback parameters optimized. Experimental results showed that the working bandwidth of the developed seismic sensor was extended to 0.005–20 Hz, validating the positive effects of the negative feedback. In addition, the developed device produced a lower self-noise level at the low frequency domain in comparison to the commercial seismic sensor of CME6011, validating its large dynamic ranges. Thus, this MEMS electrochemical seismic sensor can be potentially used in the field requesting seismic monitoring at the ultra-low frequency domain with large dynamic ranges and low noise levels.

## References

[1] Havskov J., Alguacil G. (2010). Instrumentation in Earthquake Seismology.

[2] Niu F., Silver P.G., Daley T.M., Cheng X., Majer E.L. (2008). Preseismic velocity changes observed from active source monitoring at the Parkfield SAFOD drill site. Nature.

[3] Herwijnen A.V., Schweizer J. (2011). Monitoring avalanche activity using a seismic sensor. Cold Reg. Sci. Technol..

[4] Zeng R., Lin J., Zhao Y.J. (2014). Development situation of geophones and its application in seismic array observation. Prog. Geophys..

[5] Liu Y.J. (2009). The Experiments and Studies on Broadband Electric Feedback Seismometer Based on KS-1 Pendulum (In Chinese).

[6] Zhang G., Hu S. (2010). Dynamic characteristics of moving-coil geophone with large damping. Int. J. Appl. Electromagn..

[7] Bakhoum E.G., Cheng M.H.M. (2012). Frequency-selective seismic sensor. IEEE Trans. Instrum. Meas..

[8] Kamata M. Front end fidelity for seismic acquisition. Proceedings of the 10th SEGJ International Symposium.

[9] Liang T.C., Lin Y.L. (2012). Fiber optic sensor for detection of ground vibrations. Proc. SPIE.

[10] Jaroszewicz L.R., Krajewski Z., Teisseyre K.P. (2011). Usefulness of AFORS—Autonomous fibre-optic rotational seismograph for investigation of rotational phenomena. J. Seismol..

[11] Chistyakov V.A. (2011). Portable seismic sensor. Seism. Instrum..

[12] Bertolini A., DeSalvo R., Fidecaro F., Takamori A. (2006). Monolithic folded pendulum accelerometers for seismic monitoring and active isolation systems. IEEE Geosci..

[13] Milligan D.J., Homeijer B., Walmsley R. An ultra-low noise MEMS accelerometer for seismic imaging. Proceedings of the IEEE Sensors Conference 2011.

[14] Hons M., Stewart R., Lawton D., Bertram M., Hauer G. (2008). Field data comparisons of MEMS accelerometers and analog geophones. Lead. Edge.

[15] Mougenot D., Thorburn N. (2004). MEMS-based 3C accelerometers for land seismic acquisition: Is it time?. Lead. Edge.

[16] R-Sensors LLC (Russia) Molecular-Electronic Broadband Seismometers: CME-60XX. http://r-sensors.ru/1_products/Descriptions/CME-6011.pdf.

[17] Deng T., Chen D., Wang J., Chen J., He W. (2014). A MEMS based electrochemical vibration sensor for seismic motion monitoring. J. Microelectromech. Syst..

[18] Levchenko D.G., Kuzin I.P., Safonov M.V., Sychikov V.N., Ulomov I.V., Kholopov B.V. (2010). Experience in seismic signal recording using broadband electrochemical seismic sensors. Seism. Instrum..

[19] Hurd R.M., Lane R.N. (1957). Principlis of very low power electrochemical control devices. J. Electrochem. Soc..

[20] Wittenborn A.F. (1958). Analysis of a logarithmic solion pressure detector. J. Acoust. Soc. Am..

[21] Collins J.L., Richie W.C., English G.E. (1964). Solion infrasonic microphone. J. Acoust. Soc. Am..

[22] Larcam C.W. (1965). Theoretical analysis of the solion polarized cathode acoustic linear transducer. J. Acoust. Soc. Am..

[23] Abramovich I.A., Kharlamov A.V. (2003). Electrochemical Transducers and a Method for Fabricating the Same. U.S. Patent.

[24] Huang H., Carande B., Tang R., Oiler J., Zaitsev D., Agafonov V., Yu H. (2013). A micro seismometer based on molecular electronic transducer technology for planetary exploration. Appl. Phys. Lett..

[25] He W.T., Chen D.Y., Wang J.B., Chen J., Deng T. Extending upper cutoff frequency of electrochemical seismometer by using extremely thin Su8 insulating spacers. Proceedings of the IEEE Sensors 2013.

[26] He W.T., Chen D.Y., Li G.B., Wang J.B. (2012). Low frequency electrochemical accelerometer with low noise based on MEMS Key. Eng. Mater..

[27] Sun Z.Y., Chen D.Y., Wang J.B., Chen J. A MEMS Based Electrochemical Seismometer with a Novel Integrated Sensing Unit. Proceedings of the MEMS 2016 Conference.

